# A rare morphology of the cardiac fibroma in a child: a case report

**DOI:** 10.3389/fcvm.2024.1357747

**Published:** 2024-03-27

**Authors:** Yunfei Tian, Jiayi Lin, Xiaohui Yang, Debin Zeng, Yuan Hu, Jingnan Chen, Zhongshi Wu, Xicheng Deng

**Affiliations:** ^1^ Heart Center, The Affiliated Children’s Hospital of Xiangya School of Medicine, Central South University, Changsha, China; ^2^ Department of Echocardiography and Ultrasound, The Affiliated Children's Hospital of Xiangya School of Medicine, Central South University, Changsha, China

**Keywords:** cardiac fibroma, pediatric, echocardiography, surgery, cardiac mass

## Abstract

Here we report a rare morphology of a cardiac fibroma in a child. A 2-year and 8-month-old toddler came for “chronic constipation” and was found to have a heart murmur on cardiac auscultation. Further transthoracic echocardiography suggested “a strong echogenic mass in the left ventricular wall, with some part of “a string of beads” in shape extending into left ventricle outflow tract”, which was atypical for either a tumor, thrombus or vegetation. The child underwent resection of the mass and mitral valvuloplasty. Pathological examination confirmed the mass as a cardiac fibroma.

## Introduction

**Figure 1:**
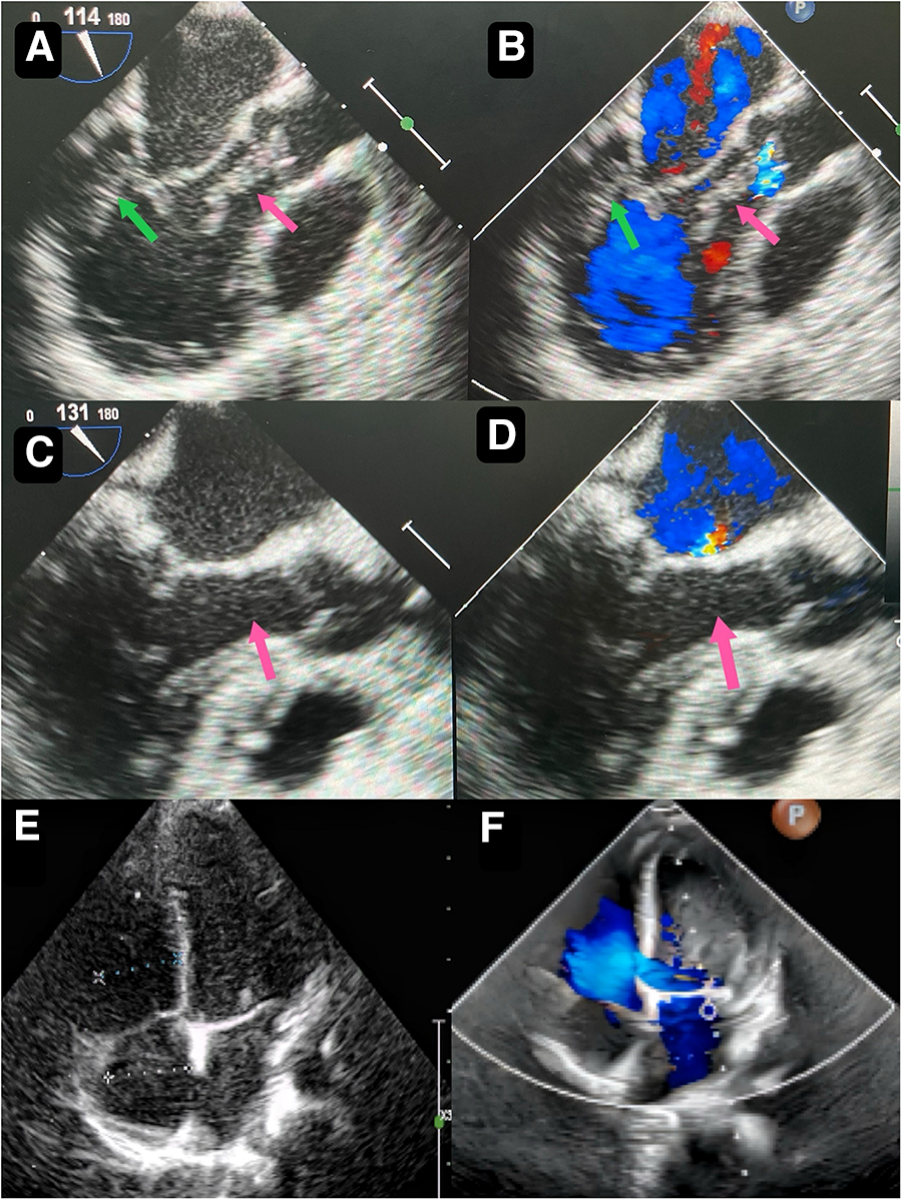
Preoperative transthoracic echocardiography reveals a slightly hyperechoic, approximately 32 mm by 16 mm mass in the posterior wall of the left ventricle (**A**) echogenic “string of beads” is observed wiggling in the left ventricular outflow tract, with one end connected to the posterior part of the left ventricle and the other end appearing to be connected to the left coronary sinus of the aorta (**B**) postoperative transesophageal echocardiogram shows complete removal of the mass (**C**) postoperative transesophageal echocardiogram shows competent mitral valve (**D**) preoperative transthoracic echocardiogram shows competent mitral valve (**E**) postoperative transthoracic echocardiogram mitral valve shows trivial regurgitation (**F**).

Primary cardiac fibromas in children are exceedingly rare and predominantly occur in infants and young children under the age of 2 (1, 2). These fibromas are typically solitary and mainly located in the left ventricle, with the right ventricle and ventricular septum being less common sites of occurrence (3). Cases may present with evident clinical symptoms and signs, though some remain asymptomatic (4). Physical examination often sees the presence of a heart murmur in symptomatic children. Echocardiography can detect homogeneous exogenic masses within the cardiac chambers (Figure 1E), while computed tomography or magnetic resonance scans can provide a more precise assessment of the tumor's location, size, number, and hemodynamic alterations (5, 6). Here we report a case of cardiac fibroma with an atypical morphology. Surgical excision and pathology confirmed it as a cardiac fibroma.

## Case report

A 2-year-and-8-month-old toddler was admitted to the hospital for evaluation of chronic constipation attributed to long-standing low-fiber diet and poor therapeutic effect of prolonged lactulose use on softening stools. A heart murmur was noted on physical examination. The child had no relevant medical history or signs of infection, trauma, cold, or any other predisposing factors. Preoperative transthoracic echocardiography revealed a hyperechoic, approximately 32 mm by 16 mm mass in the posterior wall of the left ventricle (Figure 1A). In addition, an echogenic “string of beads” was observed wiggling in the left ventricular outflow tract, with one end connected to the posterior part of the left ventricle and the other end appearing to be connected to the left coronary sinus of the aorta (Figure 1B). The sizes of the four chambers were considered normal, and the ejection fraction was 63%. Interestingly, this child showed no clinical symptoms and results of coagulation assays, neutrophil, C-reactive protein, complete antinuclear antibody, antineutrophil cytoplasmic antibodies, and antistreptolysin O titer tests were all insignificant. A diagnosis of tumor, thrombus or vegetation was yet to be made.

**Figure 2:**
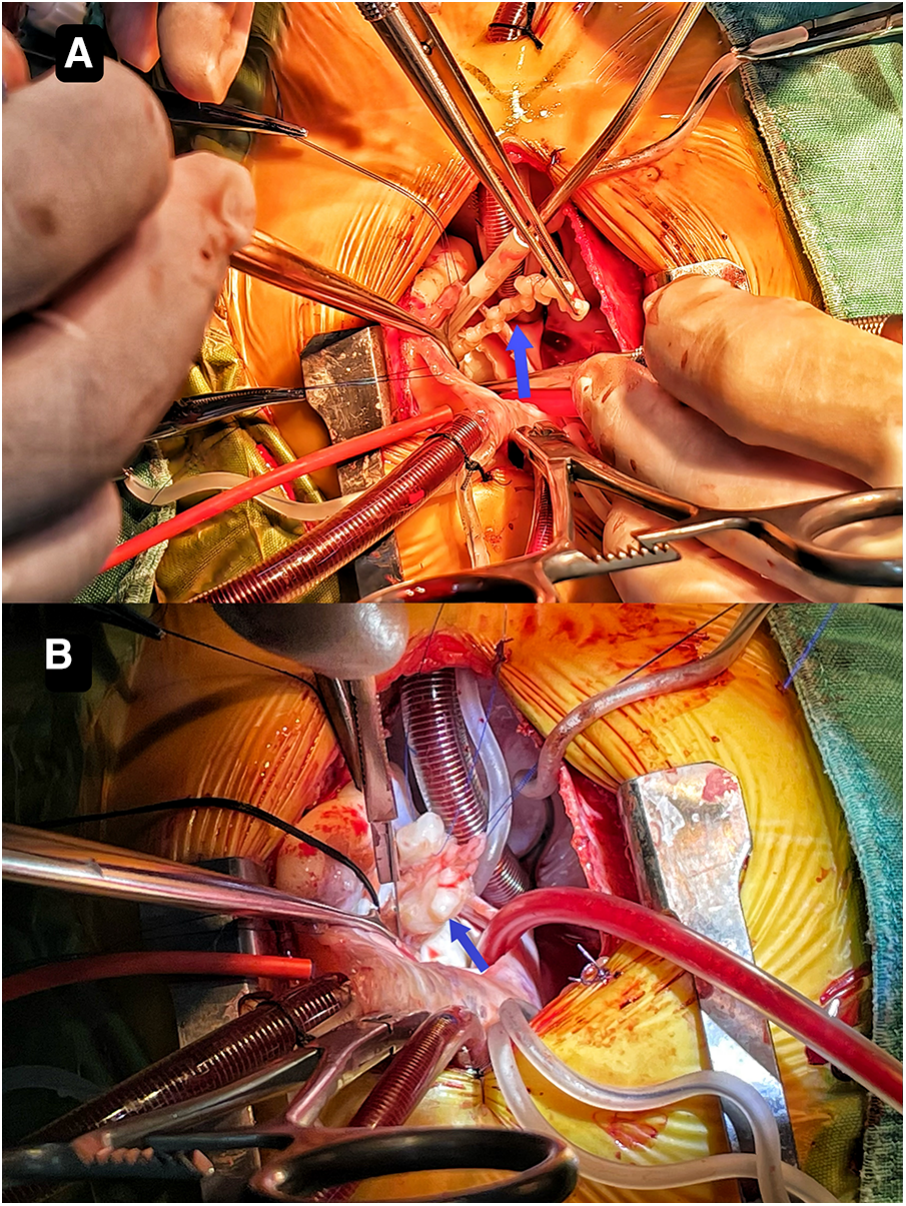
It shows that part of the mass resembles a string of beads (**A**) the other part is embedded in the posterior left ventricular wall close to the posterolateral papillary muscle (**B**).

**Figure 3:**
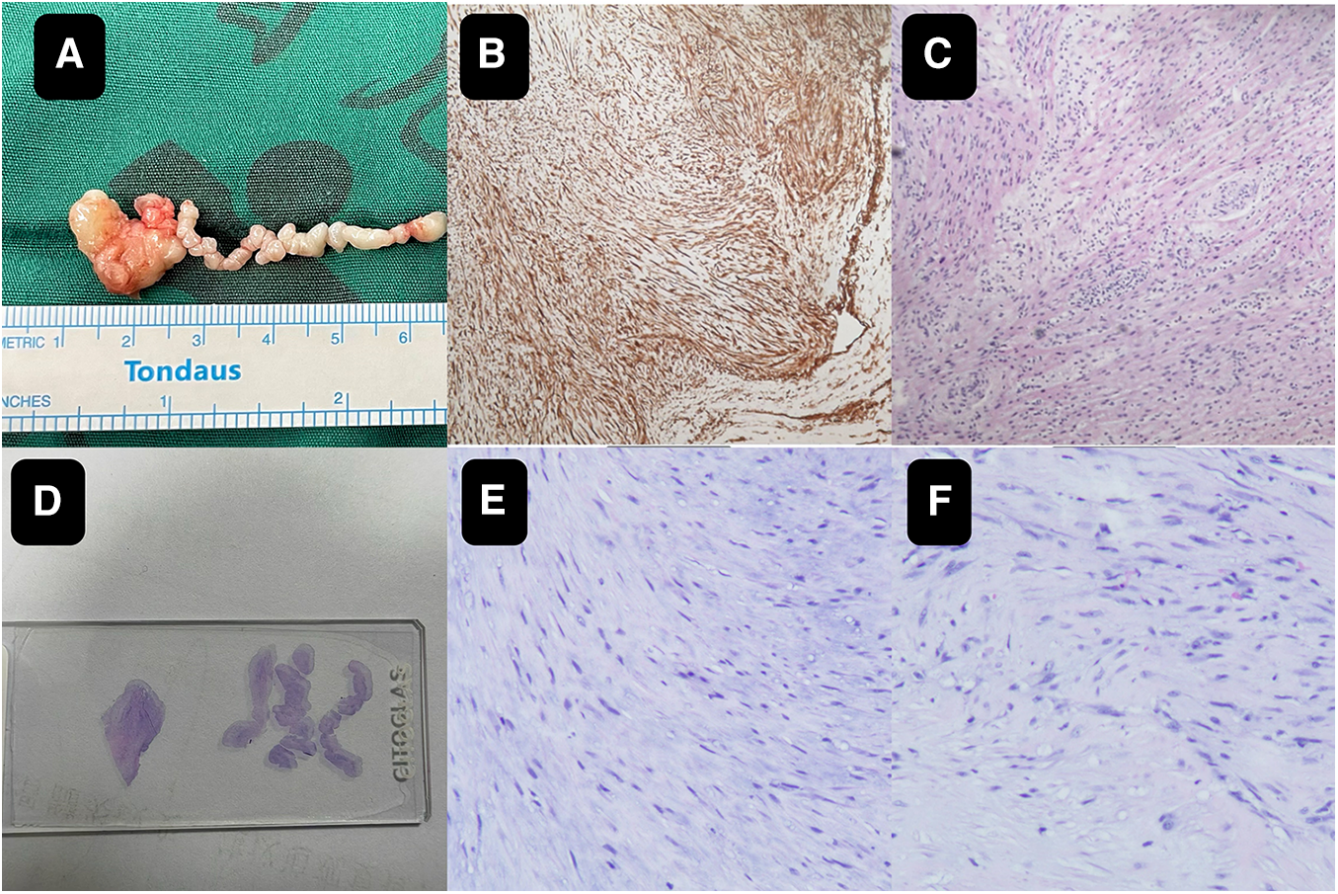
The close-up of the specimen (**A**) subsequent histopathological analysis confirmed the mass as a cardiac fibroma with myxoid degeneration (**B**,**C**). On the pathological slide it shows on the left the mass part and on the right the string part (**D**) the histology images of the wiggling string part (**E**) the histology images of the mass part (**F**).

Though the child was generally doing well and hemodynamics stable, there remained a concerning risk that the string part may break off and result in embolism. After careful consideration and discussion in a multidisciplinary team, surgical excision was planned. During the procedure, an incision was made in posterior leaflet of the mitral valve to expose the mass. It was shown that part of the mass was like a string of beads (Figure 2A), while the other part was embedded in the posterior left ventricular wall, close to the posterolateral papillary muscle (Figure 2B). The mass was predominantly white, with an intact capsule and a tough texture. After successful removal of the mass, water injection test showed significant regurgitation of the mitral valve from anterior leaflet prolapse. Mitral valve repair was performed. The ascending aortotomy was performed to exclude any residual mass in the aorta, though there was no residual mass found. Postoperative transesophageal echocardiogram showed complete removal of the mass (Figure 1C) and competent mitral valve (Figure 1D). Subsequent histopathological analysis confirmed the mass as a cardiac fibroma with myxoid degeneration (Figure 3). The results of the immunohistochemical analysis of the heart tumor specimen was as follows: Ki-67(10%+), DES (+), SMA (+), CR (focal +), CD34 (vascular +), Vim (+), EMA (−), CK (−), CD163 (+), ALK (−). The recovery was uneventful and the patient was discharged on postoperative day 10. Upon Follow-up, investigations including chest radiogram and electrocardiogram revealed no significant abnormalities. Transthoracic echocardiography demonstrated mild hyperechogenicity of the left ventricular papillary muscles, potentially related to postoperative changes. There was trivial regurgitation of the mitral valve (Figure 1F). Left ventricular systolic function was preserved. The patient's family reported no issues with daily activities or exercise tolerance.

## Discussion

Cardiac fibromas are extremely rare heart tumors, particularly in children, and typically occur between a few months to a few years of age. The clinical presentation of cardiac fibromas in children can vary greatly. Some patients may not exhibit noticeable symptoms, while others may experience symptoms such as heart murmurs, shortness of breath, and arrhythmia (7). In this particular case, the patient did not have any noticeable symptoms in her daily life, but a heart murmur was detected during physical examination when they sought medical attention for chronic constipation. The diagnosis of cardiac fibromas generally entails various imaging and laboratory tests, including echocardiography, computed tomography, and sometimes cardiac catheterization (8). Echocardiography is the most frequently employed diagnostic tool as it provides for the visualization of the location, size, and characteristics of a cardiac tumor (9, 10).

In the present case, however, as the morphological characteristics were not typical for a fibroma, and could not exclude thrombus or vegetation in this case, it was difficult to make a confirming diagnosis before surgical excision and pathology. Rhabdomyoma, which originates from cardiac fibroblasts, is a hamartoma formed during the development of heart muscle cells and accounts for more than 60% of primary heart tumors in children. It is more likely to occur in the left and right ventricular wall or septum (11). Fibroma, which is derived from connective tissue fibroblasts, is the second most common benign primary cardiac tumor in children and is more common in infants under 1 year of age. The most common location is the ventricular septum and free wall of the ventricle, rarely the atrium (12, 13). Cardiac myxoma, another common type of cardiac tumors, consists of large numbers of stellate or polygonal myxoma cells with myxoid stroma. It is the most common cardiac tumor in adults, but rare in children. It is often found in the left atrium and rarely in the heart valves and ventricles (14). Malignant cardiac tumors in children are rare, accounting for about 10% of all cardiac tumors in children. Most of these are metastatic malignancies and the incidence is 10–20 times that of primary malignancies. For thrombus, echocardiography often shows sessile masses, enlarged atria, low cardiac output. Clinical signs include congestion and response to thrombolytic therapy. For vegetations, Echocardiography often reveals vegetations with irregular mobility that are adherent to valves, findings that are highly associated with infective endocarditis (15–17).

Whatever it is, the management is typically determined by the severity of symptoms, the size and location of the mass, and the overall health condition of the patient. Surgical resection is required for cases involving severe symptoms or masses that impede heart function (18–22). The aim of surgical removal is to completely excise the mass while preserving normal heart tissue. Care must be taken in order to preserve the adjacent cardiac structures and function. The tumor may affect valvular apparatus, making valve repair necessary. However, this can be achieved with satisfactory outcome as shown in this case.

## Conclusion

In summary, cardiac fibromas are uncommon tumors of the heart, particularly in children. The diagnosis and treatment of cardiac fibromas in children require a comprehensive evaluation of symptoms, imaging, and laboratory test results. Sometimes, it may be difficult to differentiate it with other tumors, vegetation or thrombus. This case emphasizes the varying morphology of the cardiac fibroma and significance of diagnosing and surgically treating this condition in children and contributes to the overall body of knowledge and promoting additional research in this area.

## Data Availability

The original contributions presented in the study are included in the article/Supplementary Material, further inquiries can be directed to the corresponding author.
